# The Star-Nosed Mole Reveals Clues to the Molecular Basis of Mammalian Touch

**DOI:** 10.1371/journal.pone.0055001

**Published:** 2013-01-30

**Authors:** Kristin A. Gerhold, Maurizio Pellegrino, Makoto Tsunozaki, Takeshi Morita, Duncan B. Leitch, Pamela R. Tsuruda, Rachel B. Brem, Kenneth C. Catania, Diana M. Bautista

**Affiliations:** 1 Department of Molecular and Cell Biology, University of California, Berkeley, California, United States of America; 2 Helen Wills Neuroscience Institute, University of California, Berkeley, California, United States of America; 3 Department of Biological Sciences, Vanderbilt University, Nashville, Tennessee, United States of America; Yale School of Medicine, United States of America

## Abstract

Little is known about the molecular mechanisms underlying mammalian touch transduction. To identify novel candidate transducers, we examined the molecular and cellular basis of touch in one of the most sensitive tactile organs in the animal kingdom, the star of the star-nosed mole. Our findings demonstrate that the trigeminal ganglia innervating the star are enriched in tactile-sensitive neurons, resulting in a higher proportion of light touch fibers and lower proportion of nociceptors compared to the dorsal root ganglia innervating the rest of the body. We exploit this difference using transcriptome analysis of the star-nosed mole sensory ganglia to identify novel candidate mammalian touch and pain transducers. The most enriched candidates are also expressed in mouse somatosesensory ganglia, suggesting they may mediate transduction in diverse species and are not unique to moles. These findings highlight the utility of examining diverse and specialized species to address fundamental questions in mammalian biology.

## Introduction

Despite the ubiquitous importance of touch for all organisms, understanding the molecular basis of mechanosensory transduction in mammals remains a major challenge. This is due in part to the diversity of touch-sensitive neurons that are specialized to detect a variety of complex mechanical stimuli in the environment and in part to the diffuse localization of primary afferents throughout the body. Although forward genetic screens have identified a number of molecules mediating invertebrate mechanotransduction [1], we are only now beginning to uncover molecules that mediate the unique functions of touch receptors in mammals. Few mechanically-gated ion channels have been identified, ensembles of proteins may be required for normal mechanoreceptor function, and differences in transduction molecules among fiber subtypes and across species remain unknown.

**Figure 1:**
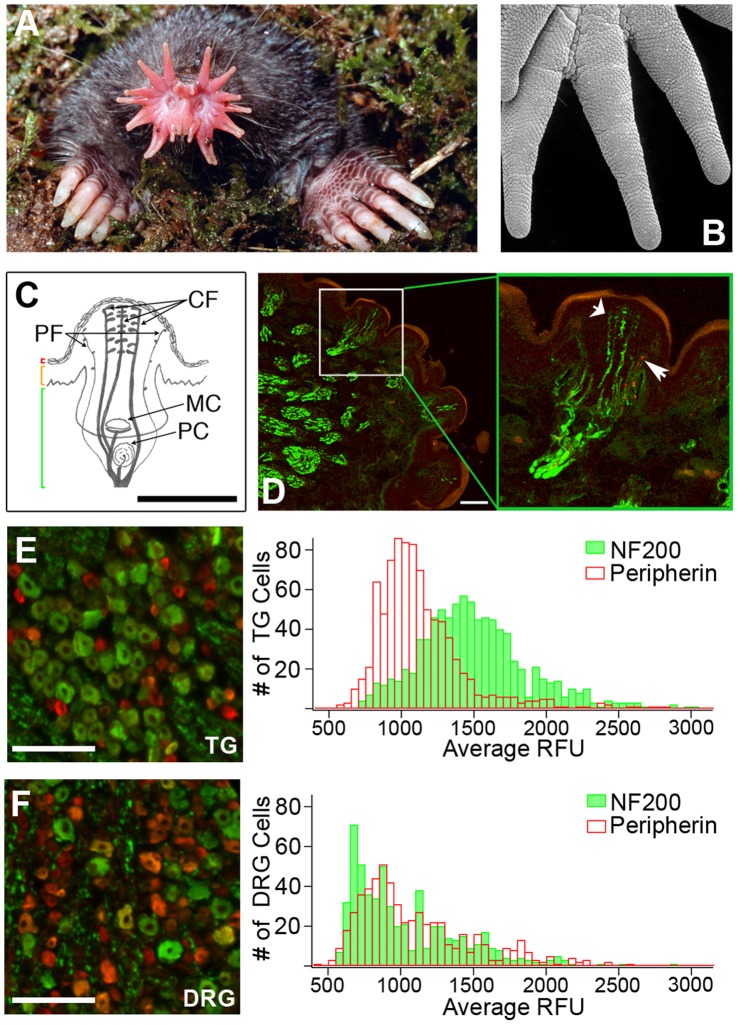
The tactile organ of the star-nosed mole is preferentially innervated by putative light touch fibers. (*A*) Image of a star-nosed mole. (*B*) EM image of the surface of the star displaying specialized Eimer’s organs. (*C*) Schematics of an Eimer’s organ in cross-section showing the column of central free nerve endings (CF) and finer peripheral free nerve endings (PF) which surround the central column. The epithelial cell layers are bracketed on the left (red = stratum corneum; orange = epidermis; green = dermis) and other mechanoreceptive complexes found in the Eimer’s organ (MC = Merkel cell, PC = Pacinian corpuscle). (*D*) Confocal images of star-nosed mole tissue stained for NF200 (green) and substance P (red). In the enlarged box, a cross-section of the star and a single Eimer’s organ. Narrow arrow indicates a substance P-positive fiber, wide arrow indicates NF200-positive fibers, scale bar = 50 µm. (*E-F*) Epifluorescence images of the TG (*E*) and DRG (*F*) stained for peripherin (red) and NF200 (green), with histograms showing the number of cells stained in one field binned by average raw fluorescence units (RFU) per cell. Scale bar = 400 µm, n = 5 sections/tissue.

Here we take a different approach to identifying candidate molecules underlying mammalian touch by exploring one of nature’s experiments in the enrichment and amplification of mechanotransduction molecules, the tactile epidermis of the star-nosed mole. Star-nosed moles (*Condylura cristata*) are renowned for the unusual mechanosensory appendages that ring their nostrils, collectively called the star (Figure 1*A*). These 22 tactile “rays” are covered with tens of thousands of domed epidermal touch organs called Eimer’s organs (Figure 1*B*). The star is only about a centimeter across, yet is innervated by over 100,000 myelinated nerve fibers, giving it the highest innervation density of any mammalian skin surface [2], [3]. Not surprisingly, the tactile receptive fields on the star are the smallest reported for any mammal, providing unparalleled tactile resolution [4]. These features make star-nosed moles a promising, if unconventional system in which to explore the molecular biology of mammalian mechanotransduction.

What are the cellular and molecular building blocks that endow the star with such high tactile sensitivity? To address this question, we characterized the subtypes of somatosensory neurons that innervate the star, identified molecules enriched in these neurons and performed functional cellular and behavioral studies. Our data show that the star organ is innervated by a large number of touch-sensitive neurons in the trigeminal ganglia (TG). The specialization of the star results in a higher number of light touch-sensitive cells in the TG versus the dorsal root ganglia (DRG), and conversely, a higher proportion of nociceptors in the DRG. We exploit this difference to identify novel candidate mammalian molecular force transducers, including cyclic nucleotide gated channels, that mediate touch and pain.

## Results

### The Star is Innervated by a Plethora of Putative Light Touch Neurons

**Figure 2:**
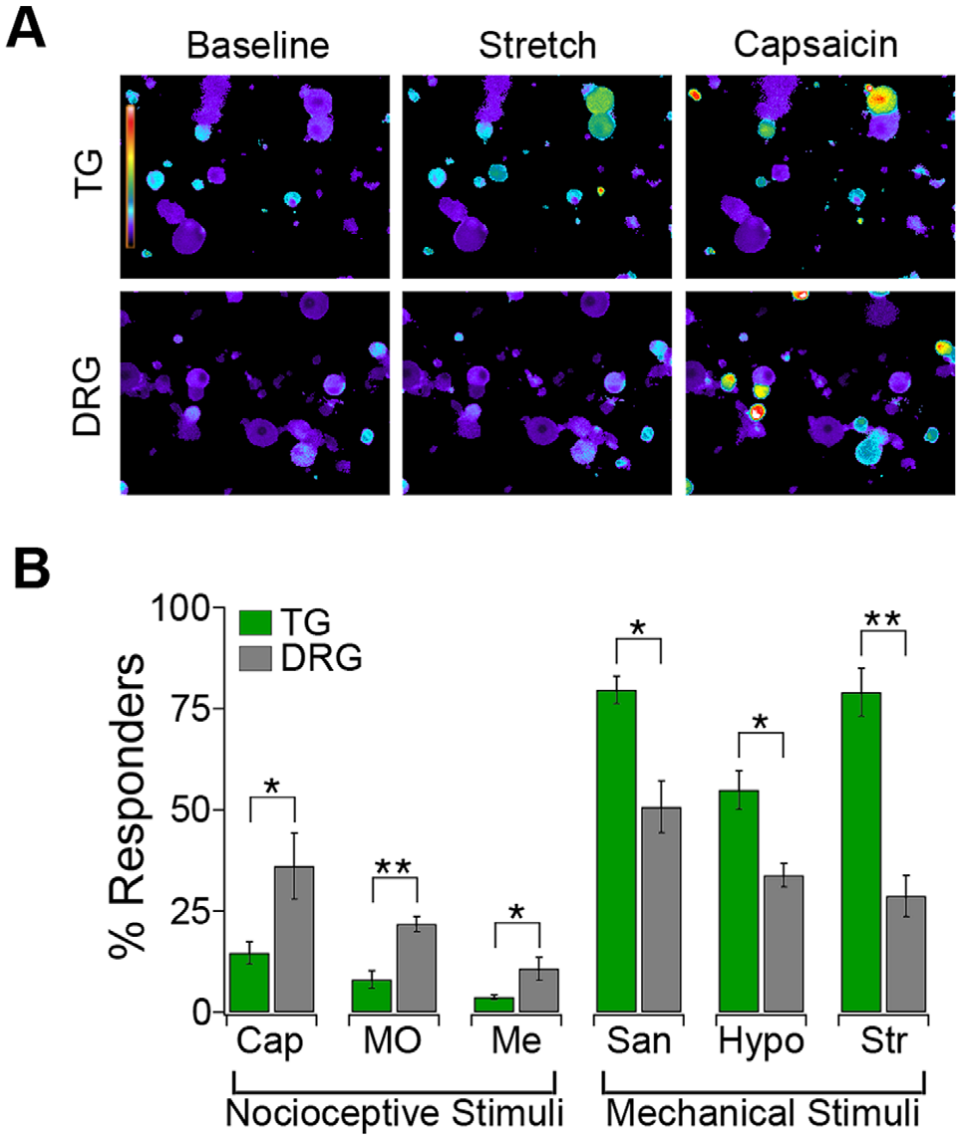
Functional enrichment of light touch-sensitive neurons in the star-nosed mole trigeminal ganglia. (*A*) Representative images of Fura-2 loaded star-nosed mole TG and DRG neurons before and after exposure to 10% radial stretch and capsaicin (1 µM). (*B*) Average percentage of TG (green) and DRG (grey) neurons activated by capsaicin (Cap), mustard oil (MO), menthol (Me), hydroxy-α-sanshool (San), hypotonic solution (Hypo) and 10% radial stretch (Str) (error bars represent s.e.m. n = 4 samples, **p< 0.01, *p< 0.05 by one way ANOVA).

We first characterized the fiber types that innervate the Eimer’s organs of the star. In mammals, the detection of noxious chemical, mechanical and thermal stimuli is thought to be mediated by unmyelinated C-fibers and lightly myelinated Aδ-fibers, both of which terminate as free nerve endings in the skin. Detection of innocuous tactile stimuli is thought to be mediated by heavily myelinated Aβ-fibers that innervate corpuscles and other specialized structures in the skin, Aδ-fibers that innervate guard hairs, and a small number of C-fibers [5]. Each Eimer’s organ contains two classical light touch receptors, a lamellated corpuscle and a Merkel cell, but is also innervated by a column of free nerve endings that terminate in the superficial epidermis (Figure 1*C*; [2]). Surprisingly, and consistent with a role in light touch detection, the majority of these fibers stained heavily for NF200 (Figure 1*D*), a marker of myelinated putative touch receptors [6], and myelin basic protein (Figure S1*A*). Sparse substance P staining, a marker of peptidergic C-fiber nociceptors [7], was observed in a few smaller fibers at the periphery of the organ (Figure 1*D*). The low levels of substance P staining are not simply due to poor antibody affinity, as the mole cornea is densely innervated by substance P-positive fibers (Figure S1*B*). Likewise and similar to other mammals [8], the glabrous skin of the hindpaw displays robust, non-overlapping substance P and NF200 staining (Figure S1*C*). These results demonstrate that the star organ is unique in that there is high-density innervation by light touch receptors and that many of the myelinated fibers terminate as free nerve endings in the epidermis.

### The Star-nosed Mole has Specialized Trigeminal Ganglia

We next asked whether the dense innervation of the star arises from robust branching of TG neurons or from an enrichment of light touch neurons originating in the TG. Preferential innervation by somatosensory fiber subtypes is observed in other tissues, such as the mouse cornea (Figure S1*D*), where the dense innervation of nociceptors is achieved by robust branching of projections from a small number of TG neurons (∼1%) in the ophthalmic track [9]. As such, there is no enrichment of nociceptors in the mouse TG (Figure S2), which contain similar numbers of myelinated light touch receptors (49.2±16.5%) and nociceptors (42.6±10.7%).

Unlike mouse sensory ganglia, immunofluoresence analysis of mole sensory ganglia showed a higher percentage of NF200-positive neurons (57.2±9.6%) compared to peripherin-positive neurons (13.4±7.4%) in the TG (Figure 1*E*). In contrast, the mole DRG displayed similar numbers of NF200- and peripherin-positive cells (36.1±6.2% vs 35.0±6.5%; Figure 1*F*). As in the mouse, the star-nosed mole NF200-positive neurons possess significantly larger somata than peripherin-positive neurons in both the TG and DRG (TG: 180.4±5.97 µm^2^ vs 105±6.29 µm^2^, p = 0.03; DRG: 234±14.1 µm^2^ vs 150±23.7 µm^2^, p = 0.04, n = 3, one-way ANOVA), a strong indication that these cells may function in light touch detection. These data reveal a novel anatomical specialization of the mole somatosensory system whereby an enrichment of light touch fibers in the star arises both from branching [2] and from an increased number of putative light touch neurons in the TG.

### The Star-nosed Mole Trigeminal Ganglion Contains More Mechanosensitive Neurons than the Dorsal Root Ganglion

These data predict that the star-nosed mole TG are functionally enriched in cells that detect innocuous mechanical stimuli while the DRG are enriched in capsaicin- and heat-sensitive neurons. Thus, we used calcium imaging to compare the activity of cultured neurons isolated from the mole TG and DRG (Figure 2*A*). We subjected neurons to capsaicin, mustard oil and menthol (Figure 2*B* *, left)*, irritants that preferentially activate nociceptors via TRPV1, TRPA1 and TRPM8, respectively [10]–[12], and examined responses to hydroxy-alpha-sanshool (sanshool), hypo-osmotic stimuli, and low-magnitude radial stretch (10%, Figure 2*B*, right), which predominantly activate light touch neurons [13]. Consistent with the antibody staining, capsaicin, mustard oil, and menthol activated a significantly higher percentage of neurons in the DRG compared to the TG (DRG: 36.1±8.2% versus TG: 14.7±2.8% for capsaicin). Conversely, hypotonic solution, sanshool and radial stretch activated a significantly higher percentage of TG neurons than DRG neurons (TG: 79.6±5.9% versus DRG: 33.86±2.9% for stretch). These results demonstrate that the star-nosed mole TG are highly specialized anatomically and functionally to mediate high tactile sensitivity.

### Using Transcriptome Profiling to Identify Candidate Touch Transducers

**Figure 3:**
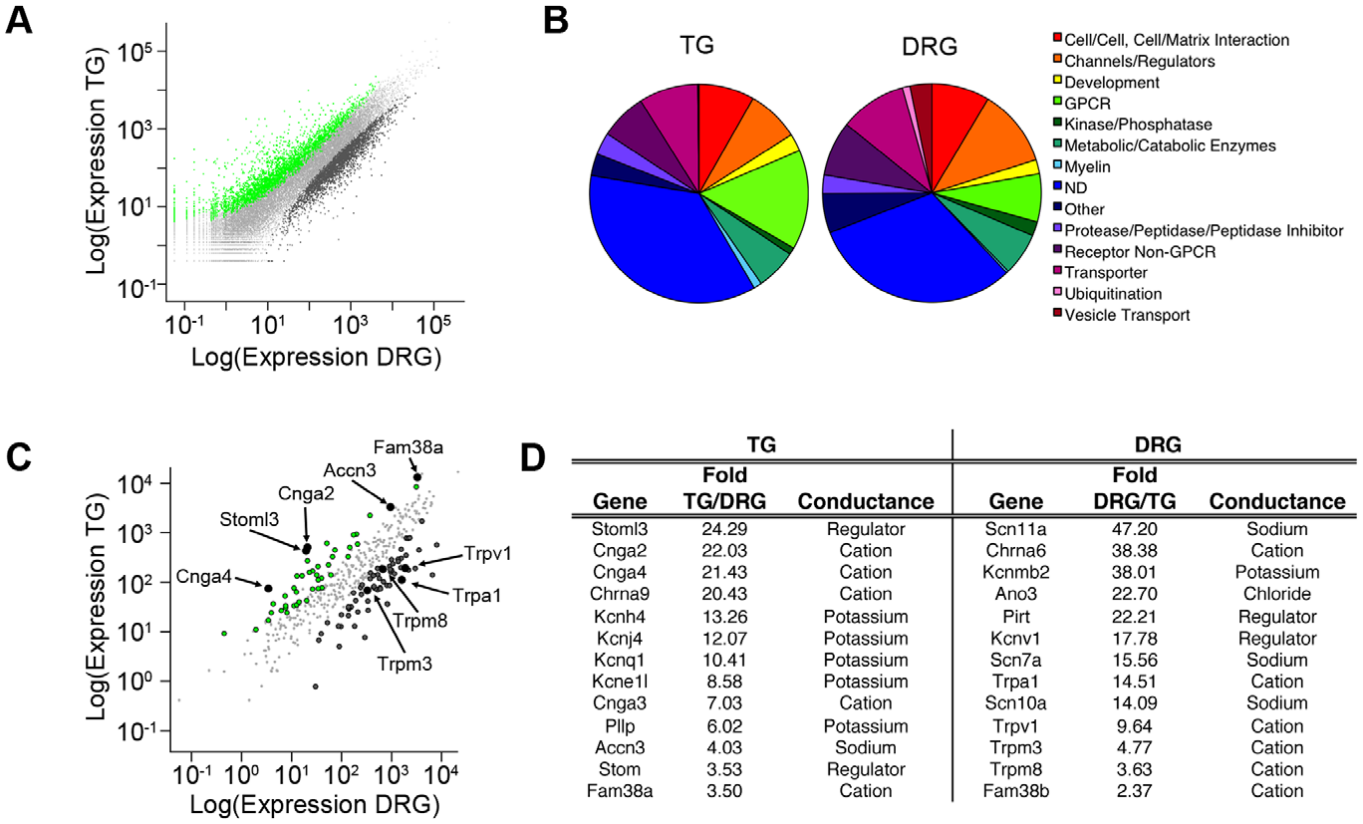
Transcriptome profiling of mole trigeminal and dorsal root ganglia. (*A)* Dot plot of expression levels of mapped genes in the star-nosed mole TG and DRG. Genes differentially expressed between TG or DRG (p_adj_< = 0.05) are shown in green and dark grey, respectively. *(B)* Gene Ontology classification of transmembrane proteins differentially expressed between the star-nosed mole TG and DRG. (*C)* Dot plot of expression levels of known ion channels in the star-nosed mole TG versus DRG. Genes differentially expressed between TG or DRG (p_adj_< = 0.05) are shown in green and dark grey, respectively. Black dots represent individual genes as labeled. (*D*) Top channels and channel regulators enriched in mole TG and DRG. This list excludes channels also differentially expressed between mouse ganglia. Table shows gene name in mouse, fold enrichment in mole, and putative ion conductance.

The enrichment of functional light touch neurons in the TG and nociceptors in the DRG suggests that these tissues vary at a molecular level. If this is indeed the case, comparing transcripts may lead to the identification of molecular players that mediate touch or pain transduction. We used mRNA-Seq to detect transcripts expressed in mole TG and DRG, and we compared expression levels between these two tissues. Currently there is no available annotated genome for the star-nosed mole, or any other member of the order Soricomorpha. As such, we mapped mRNA-Seq reads to multiple known mammalian transcriptomes iteratively. Using the Stampy algorithm [14], reads from TG and DRG were first aligned to the *Homo sapiens* transcriptome; unmapped reads were then aligned to the *Canis lupus familiaris* transcriptome, and finally to the *Mus musculus* transcriptome. This strategy successfully aligned 42%–65% of sequenced reads per sample. This is quite high, given that reads typically align at ∼87% to a well annotated genome [15]. The DESeq package [16] was employed to test for differential gene expression between the TG and DRG samples (p_adj_ < 0.05). We identified 3231 genes with significantly elevated expression in the TG and 3033 in the DRG (Figure 3*A*). We hypothesized that a number of these genes also differ between mouse TG and DRG, due to the different organ structures, localization, target organ innervation patterns, central and peripheral circuitry and support cells of these ganglia. We carried out a parallel transcriptional profiling analysis of TG and DRG in the mouse and found 51 genes that show significantly higher expression in both the mouse and mole TG compared to DRG and 72 genes more highly expressed in both mouse and mole DRG. For example, Hoxd1 is required for normal nociceptor innervation of thoracic and cervical spinal cord levels [17], and shows significantly higher expression in both mole and mouse DRG (fold change in DRG vs TG: star-nosed mole = 4.76, p_adj_ = 9.26*10^−5^, mouse = 1.5, p_adj_ = 0.034).

To identify transduction molecules, we limited our analyses to predicted membrane proteins that were not differentially expressed between mouse TG and DRG, and were more highly expressed in mole TG (788 genes) or DRG (1159 genes). The genes belong to a wide variety of classes as verified by Gene Ontology analysis (Figure *3B*). Consistent with a higher percentage of large diameter myelinated neurons, the SNM TG showed increased transcript numbers for a larger number of myelin specific transmembrane proteins compared to DRG. Conversely, DRG showed higher expression of a larger number of proteins involved in vesicle trafficking and exocytosis, consistent with an increased number of peptide releasing nociceptors.

In mechanosensory cells, ion channels underlie the transduction of mechanical stimuli into electrical signals. We therefore focused on genes that encode ion channels or channel modulators and are significantly enriched in the DRG or TG (Figure 3*C–D*). The DRG-enriched genes include a number of channels implicated in nociception including: the tetrodotoxin-insensitive sodium channels Nav1.8 (Scn10a) and Nav1.9 (Scn11a; [18], [19]), the cold-sensitive ion channel Trpm8 [10], the heat-sensitive channel Trpm3 [20], the irritant receptor Trpa1 [11], [21], the heat- and capsaicin-receptor Trpv1 [22] and the putative mechanosensitive channel Piezo2 (Fam38b; [23], [24]).

Several ion channels previously implicated in mechanotransduction were enriched in the TG, including: ASIC3 (Accn3), a homologue of the MEC4/10 channels which transduce mechanosensitive currents in *Caenorhabditis elegans* [25], Piezo1 (Fam38a), a mechanosensitive channel expressed in mouse kidney, lung and skin [23], Cnga2, a cyclic-nucleotide-gated channel required for mechanical- and odorant-evoked responses in olfactory neurons [26], and Cnga3 which plays a role in inflammatory hypersensitivity to thermal and mechanical stimuli [27]. In addition, we observed significantly higher expression in the TG of Stoml3 and Stom, homologues of the *Caenorhabditis elegans mec-2* gene that modulates mechanosensitive channels [28]; both of these proteins have also been implicated in light touch detection in mammals [29]. These data show that by comparing the star-nosed mole DRG and TG transcriptome we can classify channels and signaling molecules as either pain, or light touch candidate transducers.

**Figure 4:**
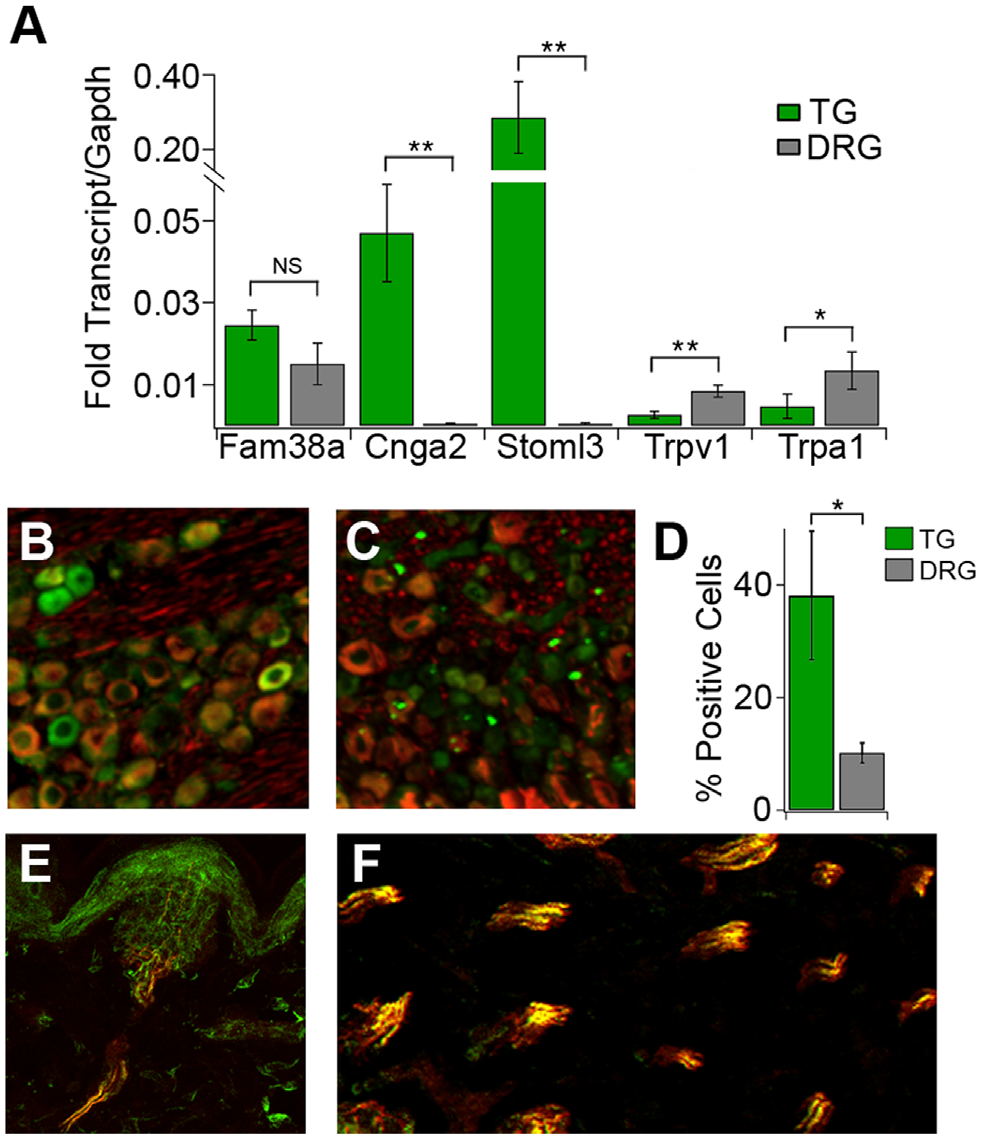
Expression analysis of candidate touch and pain transducers. (*A*) qPCR analysis of selected transcripts in TG (green) and DRG (grey). Results show average expression normalized to Gapdh (n = 3 samples/tissue). (B-F) Images of star-nosed mole tissue stained with antibody against CNGA2 (green) and NF200 (red). (*B*) Trigeminal ganglia (TG) show stronger staining for CNGA2 than (*C*) dorsal root ganglia (DRG). (*D*) Percentage of total neurons stained positive for CNGA2 in TG (green) and DRG (grey) n = 4 sections/tissue. (*E*) Cross section of a single Eimer’s organ and (*F*) nerve tracts innervating multiple Eimer’s organs show strong CNGA2 staining and high overlap with NF200 staining. Error bars represent s.e.m. **p< 0.01, *p< 0.05, NS = p>0.05 by one way ANOVA).

To validate our high-throughput expression analysis, we performed quantitative PCR (qPCR) on several of the signaling molecules enriched in the TG and DRG (Figure 4*A*). As expected, qPCR of Fam38a, Cnga2 and Stoml3 showed higher expression in the TG, while Trpa1 and Trpv1 were more highly expressed in the DRG, though differences in Fam38a expression were not significant. We also analyzed expression of CNGA2 protein in mole sensory ganglia and the star. Consistent with a role in light touch detection, and our transcriptome profiling, immunofluorescence analysis of mole sensory ganglia stained with antibodies against CNGA2 showed a higher percentage of CNGA2-positive neurons in the TG as compared to the mole DRG (Figure 4 *B–D*); these cells also displayed robust NF200 immunoreactivity. To determine if Cnga2 is expressed in mole TG neurons that project to the star, we examined Cnga2 staining in the fibers that innervate the star. Indeed, Eimer’s organs are densely innervated by CNGA2-positive fibers, the vast majority (>90%) of which are also NF200-positive (Figure 4*E–F*). These data show that our transcriptome expression analysis can indeed identify novel candidate transducers that are enriched in light touch neurons of the star-nosed mole.

### Candidate Touch Transducers in the Mouse Somatosensory System

**Figure 5:**
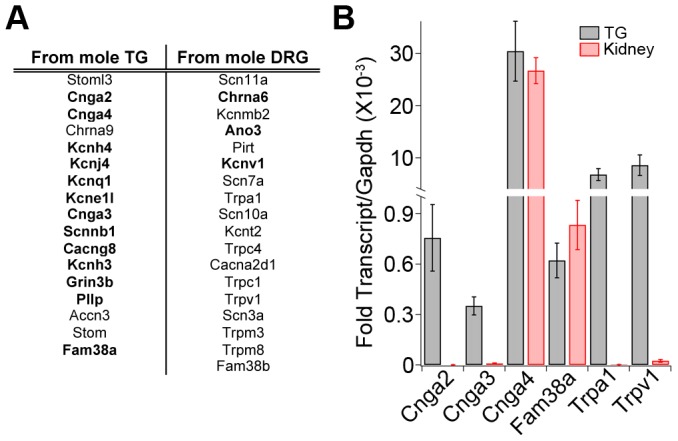
Expression of candidate transducers in mouse ganglia. *(A)* Ion channels enriched in mole TG and DRG that were amplified by RT-PCR from mouse TG and DRG. All genes were amplified independently from TG and DRG samples isolated from two mice. Channels shown in bold are candidates that have not been previously reported as expressed in somatosensory neurons. (B) qPCR analysis of selected genes in mouse TG and kidney. Results show average expression normalized to Gapdh (n = 3). Error bars represent s.e.m.

Definitive evidence for the involvement of a gene in mechanotransduction must come from functional assays. Unfortunately, such assays are not possible in star-nosed moles. We thus examined expression of the most TG- and DRG-enriched channels in mouse sensory ganglia. ∼85% of these channels were detected in mouse TG and DRG by RT-PCR (Figure 5*A*). Many of these channels have not been previously implicated in mammalian somatosensory transduction. qPCR of mouse ganglia also demonstrated expression of the TG-enriched ion channels Cnga2, Cnga3, Cnga4, and Fam38a, as well as the DRG-enriched TRPA1 and TRPV1 (Figure 5*B*). Overall, these channels represent excellent candidates to probe in the more tractable mouse model.

## Discussion

Despite intensive study in recent years, the molecular basis of mechanotransduction remains poorly understood and few mechanically gated ion channels have been identified. Here we take a new approach by investigating the hypertrophied mechanosensory epidermis of the star-nosed mole. We showed that the dense innervation of the star by myelinated light touch fibers originates from a specialized trigeminal system. In general, the mammalian somatosensory system is broadly tuned to detect a diverse array of innocuous and noxious stimuli. Unlike other mammalian somatosensory ganglia, or even the star-nosed mole DRG that innervate the body, the star-nosed mole TG contain relatively few classical nociceptors (Figure 1*E–F*, 2*B*). Thus, the high tactile acuity of the star may arise at the expense of thermal and chemical sensitivity, as the lack of peptidergic C-fibers and nociceptive molecules (*e.g.* TRPA1 and TRPV1) may affect the ability of the star to detect irritants and other noxious stimuli. Consistent with this, topical application of capsaicin to the hindpaw elicits nocifensive responses similar to those reported in rodents, but application to the star evokes no obvious behaviors (n = 3 animals).

The star-nosed mole has adapted a new strategy for achieving high tactile acuity, namely a cellular and molecular specialization of the trigeminal ganglia that innervate the star. We exploited this specialization to identify molecules enriched in the DRG and TG. Transcript analysis offers an unbiased approach to identify genes of interest, but is usually restricted to organisms for which genome information is widely available. Since there is no annotated genome data for the star-nosed mole, or any close relative, we used iterative mapping to assign the identity of reads. The use of this approach to identify novel players in touch and pain is validated by the differential expression of molecules already known to play keys roles in pain transduction in the DRG, and genes implicated in innocuous touch transduction in the TG. For example, TRPV1 in nociceptors and Stoml3 in touch neurons.

RNASeq analysis revealed an enrichment of nociceptive transcripts in the mole DRG versus the TG. For example, TRPV1 expression is ∼10-fold higher in the mole DRG than the TG and Cnga2 levels are ∼20-fold higher in the mole TG versus DRG (Figure 3*D*). Some of this enrichment can be accounted for by the varying numbers of neuronal subtypes in the DRG and TG as measured by functional imaging and histology. However, while we see 10–20 fold differences in transcript levels between the DRG and TG, the number of TRPV1- or Cnga2-positive cells only varies by ∼3-fold (Figure 2*B*, 4*D*). These findings suggest that expression differences between ganglia may arise from both a shift in the number of nociceptors, as well as expression differences within any given neuronal subtype.

We identified a number of novel candidate genes enriched in mole TG that may mediate mammalian somatosensory mechanotransduction. Cnga2 and Cnga4, the most highly enriched channels in the mole TG, play an important role in signal transduction in both the visual and olfactory systems [30]. Cnga2 is proposed to mediate both chemosensory and mechanosensory responses evoked by breathing in the mouse olfactory system [26]. If CNG channels play a role in somatosensory transduction, other components of the cyclic nucleotide pathway should be expressed in sensory neurons. This is indeed the case, as mouse sensory ganglia also express two additional genes required for cAMP signaling and olfactory transduction and behaviors in mice, the G protein (Gnal) and adenylate cyclase 3 (Adcy3) (data not shown; [31], [32]). These data suggest that cyclic nucleotide signaling may represent a new signaling pathway in touch transduction. More broadly, these findings suggest that transduction mechanisms may be conserved between divergent sensory systems. Indeed, transcriptional analysis of the *Droshophila* Johnston’s organ recently identified a number of chemo- and photo-transduction molecules that also play a role in auditory transduction [33].

Fam38a (Piezo1) is an ion channel that induces mechanically-evoked responses in many different cell types. Previous studies showed high expression in mouse kidney, lung and skin, but relatively low expression in sensory ganglia. Our RNASeq and qPCR data shows expression in mole TG and DRG. This led us to re-examine expression of Fam38a in mouse sensory ganglia. Surprisingly, we amplified Fam38a from both mouse TG and DRG and observed comparable levels of expression in mouse TG and kidney (Figure 5*B*). Likewise, RNASeq of mouse ganglia also shows expression of Fam38a, suggesting that this protein may play a role in mediating light touch transduction in mice.

Finally, we identified a number of novel, uncharacterized genes enriched in TG and DRG that contain one or more transmembrane domains and that were not previously classified as organellar. These genes, along with CNG channels and Piezo1, may participate in different aspects of transduction, acting as detectors, modulators and/or amplifiers of mechanical stimuli. Each subtype of light touch receptor has distinct mechanical thresholds, sensory adaptation properties and action potential waveforms, and it is predicted that such diversity is driven by a unique repertoire of force transducers. Thus, future studies will determine if these genes are expressed in distinct subsets of somatosensory neurons, and what role they play in detecting diverse somatosensory stimuli *in vivo*.

Our results emphasize the utility of examining both traditional model organisms and less common species that may provide important clues to sensory system function. August Krogh articulated the principle that “for such a large number of problems there will be some animal of choice, or a few such animals, on which it can be most conveniently studied” [34]. In the spirit of this approach, we exploited the high tactile acuity of the star-nosed mole to identify a multitude of candidate molecules that may mediate innocuous and noxious somatosensory stimuli. The function of these molecules can now be probed in more traditional model organisms.

## Materials and Methods

### Animals

Star-nosed moles were collected in Potter County, Pennsylvania, under permit COL00087. Wild type adult female mice C57/Bl6 (Jackson Labs) were used for sectioning, RT-PCR and qPCR. All procedures followed guidelines for the care and use of laboratory animals from the National Institutes of Health and were approved by the Vanderbilt University Institutional Animal Care and Use Committee and the UC Berkeley Animal Care and Use Committee.

### Immunohistochemistry

Five star-nosed moles and two mice were anesthetized with 250 mg/kg i.p. injection of sodium pentobarbital and perfused with 1X PBS (pH 7.3) followed by 4% paraformaldehyde (PFA) in PBS. Trigeminal ganglia, dorsal root ganglia, star tissue, hindpaw skin and cornea were collected. Tissue was post-fixed for >4 hours in 4% PFA and incubated overnight in 20% sucrose at 4°C. Trigeminal ganglia, dorsal root ganglia, hindpaw skin and star were embedded in Tissue-Tek optimal, sectioned at 10 or 15 µm, and mounted onto glass coverslips for staining.

Sections were blocked for 90 minutes in PBST supplemented with 10% normal goat serum and incubated overnight at 4°C with primary antibody. Primary antibodies were diluted as follows: mouse anti-NF200 (Sigma) and rabbit anti-peripherin 1∶1000 (Millipore), guinea pig anti-substance P 1∶700, rabbit anti-S100 1:300 (ThermoFisher), rabbit anti-CNGA2 1:500 (Alomone labs). Sections were washed and incubated for one hour at room temperature in 1∶1000 secondary antibody. Secondary antibodies (Invitrogen) were used as follows: anti-mouse Alexa 488 or 568 for NF200, anti-rabbit Alexa 568 for peripherin, anti-guinea pig Alexa 568 for substance P, Alexa 488 anti-rabbit for S100, and Alexa 488 anti-rabbit for CNGA2. Intact corneas were stained directly after sucrose incubation.

### Imaging

Fluorescence images were captured using an inverted fluorescence microscope (IX71, Olympus), illuminated with a xenon light source (Lambda LS-xl, Sutter) with a FITC excitation and emission set for Alexa 488 (Chroma) and Texas Red excitation and emission set for Alexa 568 (Chroma). Images were captured using MetaMorph (Molecular Devices). Confocal images were collected using Leica TCS SL confocal microscope. Summation projections were compiled from a series of 8 images (1 µM Z-steps, MetaMorph).

### Quantification

For determining percentages of positive cells, cell boundaries were determined by hand using bright field images and the average intensity of each region was calculated (MetaExpress). Cells were defined as being positive for a specific marker if their intensity was more than 30% above background.

### Cell Culture

DRG and TG cells were cultured as previously described [12]. Briefly, star-nosed moles were euthanized and dorsal root ganglia and trigeminal ganglia were dissected. Ganglia were digested for 20 minutes in 7 mg/mL collagenase P (Roche) then for 2 minutes in 0.25% trypsin. Cells were dissociated manually by trituration and plated on glass coverslides coated with 1 mg/mL Poly-D-Lysine and 0.1 mg/mL laminin. Cells were cultured overnight at 37°C before imaging.

### Calcium Imaging

Calcium imaging was performed as previously described for mouse neurons [10]. Cells were loaded for an hour at room temperature with Fura-2AM (10 µM, Invitrogen) in Ringer’s solution (140 mM NaCl, 5 mM KCl, 2 mM CaCl_2_, 1 mM MgCl_2_, 10 mM D-glucose, 10 mM HEPES) supplemented with 0.2% pluronic acid (Invitrogen). Ratiometric calcium imaging was performed using alternating 340 nm and 380 nm excitation filters with images collected every three seconds. Responsive cells were defined as those whose calcium concentration increased more than 10% above the baseline after application of the stimulus.

### RNA Extraction

Star nosed mole or mouse tissues were removed and homogenized in Trizol (Invitrogen). Total RNA was extracted as per the manufacturer’s instructions. Total RNA was either DNAsed, re-purified (RNAeasy, Quiagen) and reverse transcribed (SuperScriptIII, Invitrogen) for qPCR or RT-PCR, or further purified with two rounds of poly-A selection (Dynabeads mRNA purification Kit, Invitrogen) for sequencing.

### Illumina Sequencing

Poly-A selected mRNA from trigeminal ganglia and dorsal root ganglia were fragmented using Fragmentation Reagents (Applied Biosystems). Libraries were further prepared using the Genomic Sample Prep Kit following a modified RNA-seq protocol [35] or the TruSeq kit (Illumina). One to three lanes of each tissue’s library were sequenced on a Genome Analyzer II or HighSeq 200 using the 76, 50 or 36 bp single-end sequencing protocol at the Vincent J. Coates Genomics Sequencing Laboratory at UC Berkeley.

### Transcriptome Analysis

Three (mole) or two (mouse) TG and DRG were used. All samples were derived from single individuals, except one mole DRG, combined from two individuals. Mole reads were mapped as described in the text. Mouse reads were aligned to *Mus musculus* mRNA sequences. *Homo sapiens* database: Nov 25^th^ 2010, genome.ucsc.edu. *Canis lupus familiaris* and *Mus musculus*: Nov28^th^ 2010, ncbi.nlm.nih.gov. Alignments were performed using Stampy v1.0.11 [14] with a substitution rate = 0.03. The read count for a given gene was the sum of reads aligning to the transcript(s) assigned to an entry in the Entrez Gene database (ncbi.nlm.nih.gov, Jan 2011). For mole genes, read count was the sum of all reads aligning to the homologs of that gene in the three organisms. Homology was based on the HomoloGene database (build 64) and manual annotation. Differential expression was assessed using the DESeq package for R [16] which corrected for multiple comparisons (p_adj_).

### Transmembrane Domain Prediction

Transmembrane domains were identified by removing signal peptides predicted with SignalP3.0 [36], and by analyzing the remaining sequence with SOSUI [37], TMHMM2.0 [38], and TMpred [39] programs. A protein was considered having a transmembrane domain if at least two of the three programs predicted at least one transmembrane region.

### qPCR

RNA was extracted from 3 moles as described above. qPCR was performed using SYBR GreenER (Invitrogen) on a StepOnePlus™ Real-Time PCR system (Applied Biosystems). qPCR reactions were run in 10 µL using 1 µL of product from the RT reaction with the primers listed below. CT values were calculated as the average of 2 wells. Fold expression for each was calculated averaging normalized CT values from two runs, assuming 100% efficiency. Final values were calculated as an average of the biological replicates for each tissue type.

Fam38a: Fwd TCTTCCTCTTCCAGGGGTTC, Rev ACCAGGGACATGAAGAGCAG

Cnga2: Fwd CTGGAGACCAAGATGAAGCAGA, Rev ATTACGAAGACGGAGCCCTACA

Stoml3: Fwd GAGTCACCCATAGCCCTCCA, Rev GACCACCCATGCCCTCTAGT

Trpv1: Fwd TGAAGACCTTGTTTGTGGACAG, Rev CGGGTGTAGTAGAGCATGTTGG

Trpa1: Fwd CTTATTGGTTTGGCAGTTGGTG, Rev CGGTTGGGGTAGACAGTGATG

Gapdh: Fwd CACGGCCACCCAGAAGAC, Rev TCAGATCCACGACGGACAC.

### RT-PCR

Trigeminal ganglia, dorsal root ganglia or a mix of tissues (liver, heart, lungs, kidneys, pancreas and brain) were extracted and cDNA was produced as described above with or without reverse transcriptase (no RT control). PCR was performed on 1 µL of product using Phusion Taq polymerase (NEB) and the primers described in Table S1.

### Statistics

All values are presented as average ± standard error of the mean (s.e.m.). Unless otherwise noted, all p-values were calculated using a one-way ANOVA with a Tukey’s Post Hoc analysis.

## Supporting Information

Figure S1 **Star-nosed mole NF200-positive fibers display similar characteristics to those described in mouse.** (*A*) All NF200-positive fibers (green) are myelin basic protein-positive (red). Epifluorescence image of a section through a bundle of fibers projecting through the nose. (*B*) Mole cornea shows strong substance P staining. Confocal image of the mole cornea with substance P (red) and NF200 (green), scale bar = 20 µm. (*C*) Mole hindpaw shows robust staining of both substance P (red) and NF200 (green). Confocal image, scale bar = 50 µm. (*D*) Mouse cornea shows similar staining to mole cornea. Confocal image, scale bar = 20 µm.
(TIF)

Figure S2 **Mouse TG and DRG show similar staining for peripherin and NF200.** Peripherin (red) and NF200 (green) staining of the TG (*A*) and DRG (*B*). (*C*) Quantification of the percent of NF200 and peripherin positive cells in these tissues (NS = p>0.1; n = 3 sections, error bars represent s.e.m.).
(TIF)

Table S1 **Primers for amplification of star-nosed mole enriched genes in mouse.**
(DOCX)
