# Supplementary figures and images for: The Star-Nosed Mole Reveals Clues to the Molecular Basis of Mammalian Touch
Source: PLoS One. 2013 Jan 30;8(1):e55001. doi: 10.1371/journal.pone.0055001 (PMC3559429; doi:10.1371/journal.pone.0055001)

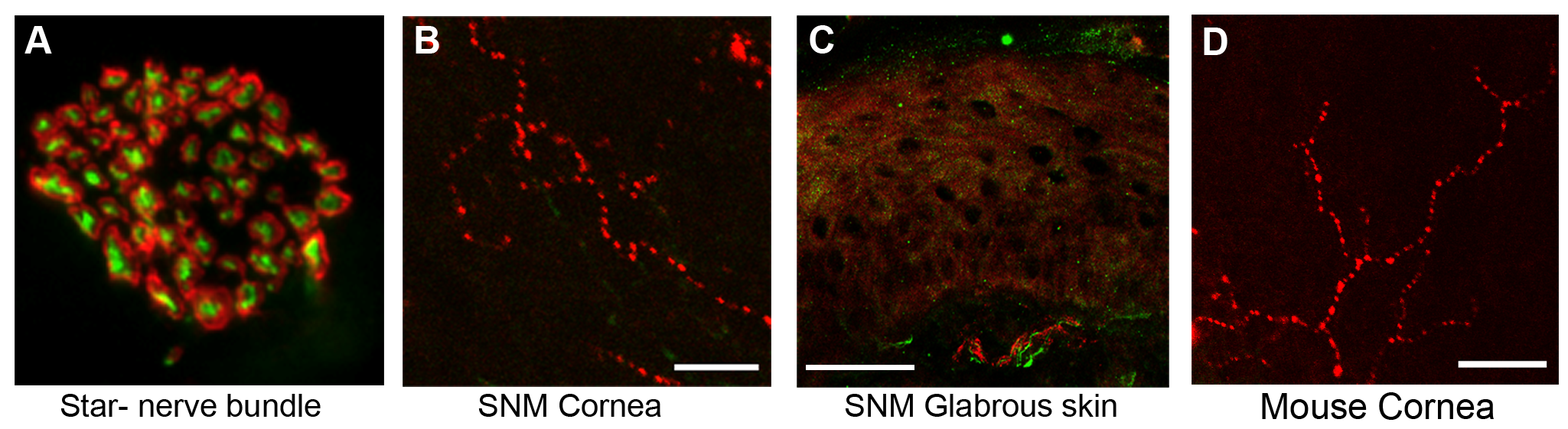

Supplement: Figure S1 — Star-nosed mole NF200-positive fibers display similar characteristics to those described in mouse. (A) All NF200-positive fibers (green) are myelin basic protein-positive (red). Epifluorescence image of a section through a bundle of fibers projecting through the nose. (B) Mole cornea shows strong substance P staining. Confocal image of the mole cornea with substance P (red) and NF200 (green), scale bar = 20 µm. (C) Mole hindpaw shows robust staining of both substance P (red) and NF200 (green). Confocal image, scale bar = 50 µm. (D) Mouse cornea shows similar staining to mole cornea. Confocal image, scale bar = 20 µm. (TIF) [file pone.0055001.s001.tif]

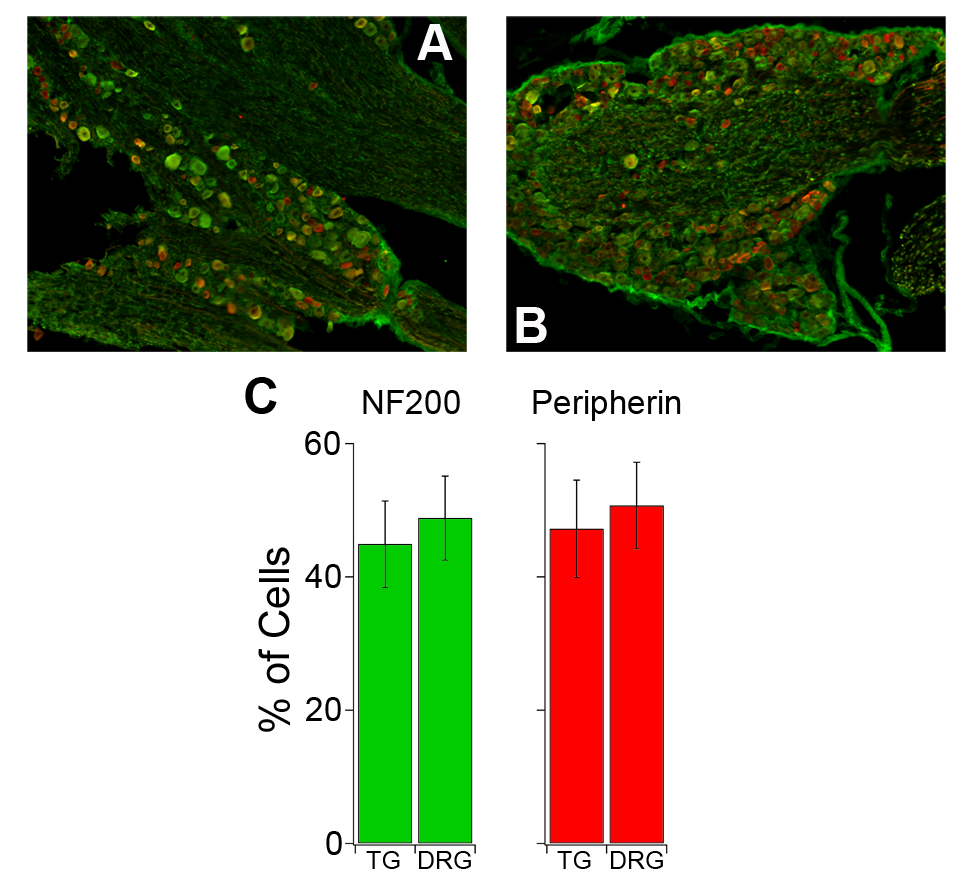

Supplement: Figure S2 — Mouse TG and DRG show similar staining for peripherin and NF200. Peripherin (red) and NF200 (green) staining of the TG (A) and DRG (B). (C) Quantification of the percent of NF200 and peripherin positive cells in these tissues (NS = p>0.1; n = 3 sections, error bars represent s.e.m.). (TIF) [file pone.0055001.s002.tif]
