# Supplementary material for: The Star-Nosed Mole Reveals Clues to the Molecular Basis of Mammalian Touch
Source: PLoS One. 2013 Jan 30;8(1):e55001. doi: 10.1371/journal.pone.0055001 (PMC3559429; doi:10.1371/journal.pone.0055001)
Supplement: Table S1 — Primers for amplification of star-nosed mole enriched genes in mouse. (DOCX) [file pone.0055001.s003.docx]

**Table S1.** Primers for amplification of star-nosed mole enriched genes in mouse.

| **Gene** | **Forward Primer** | **Reverse Primer** |
| --- | --- | --- |
| Stoml3 | CGTTACCTTCCCAATCTCAG | ACATCTTTGATTTCCACCCG |
| Cnga2 | GGCGAGGGAAAGGGCACCAA | AAGCCTGTGCGCAGCCGAAT |
| Cnga4 | GGAACATGTCTGGAAACCGA | CTCCATCCTTGGACGTTCCTT |
| Chrna9 | GTGGGTGAGAGCTGCCTTAG | ACCAACCCACTCCTCCTCTT |
| Kcnh4 | GACCTCCTTCTGTGCTCCTG | AGACGAGGAGACCGTGAGAA |
| Kcnj4 | TAGTGCATGAAATCGACGAG | TCTTCCTCCTGGCTCATAAG |
| Kcnq1 | CTCCTGTTTCTCTGTCTTCG | GAAATGGGGTGTGCTTACTT |
| Kcne1l | AACTCTGGGGCCGTTTAACT | ATGACTGGCCCTGAATGAAG |
| Cnga3 | TTGGACTACTCTGCAGATGT | AAAGTAGATGCAGGCATTCC |
| Scnn1b | AGTCCTGCAATGACACCCAG | GCTGGAAGCCAAAGTTGGTG |
| Cacng8 | GAGAGGGGTTTGTGGTGTGAA | GATGGCGCTCAGGATAGGAA |
| Kcnh3 | TGAAGCAAGAAACACAGACA | GTCTGAAGTGGAGGTCCATA |
| Grin3b | GCTGCAGTATTGGCTTCACA | GCCGTGTAGTTCCTCAGCTC |
| Pllp | AGTTCCCGTCGAAAGTGA | AGGCGATAAAGGCAGTAATG |
| Catsper1 | AGGCGCCTGGTACATCATAC | CGCTGCTTCACTGTCATGTT |
| Accn3 | TTTCACTCGAATGGGGCAAT | GAACCAGTACATTCTCCCCAGAG |
| Stom | TGCCTCGTTCTTTTTCGTAA | TTTGAGACAGGTTCTTGGTG |
| Fam38a | GCAGCCAGATGAGGAAGAGG | TAGCTCCTCCTCCAGCTCCA |
| Scn11a | TGAGGCAACACTACTTCACCAATG | AGCCAGAAACCAAGGTACTAATGATG |
| Chrna6 | TGGAACTGTGGGGCTATTTC | AGCAGGAGCTTCGGATACAA |
| Kcnmb2 | GTCAGGGCCAGCTATCATGT | TTAAAACCAACCCCCTTTCC |
| Ano3 | GAGGAGGAAACACTTCGCCC | TGCCAGTGCTGTTTGACGAA |
| Pirt | CCAAGGCCCTGGAGGTAGAC | ACAGGAAGGCAGGCCCTATC |
| Kcnv1 | ATTGCGCTCACTTGGGATGA | AGAGCTTCACGCTGTCTCAC |
| Scn7a | ACCTTACCCACTGTGAGCGT | CAGGGCCCGGTACTGTTTTT |
| Trpa1 | GGAGCAGACATCAACAGCAC | GCAGGGGCGACTTCTTATC |
| Scn10a | ACCGACAATCAGAGCGAGGAG | ACAGACTAGAAATGGACAGAATCACC |
| Kcnt2 | AAGGCTGAGCAGAAAAGGGC | AGGACAGTGGATCTGGTCGG |
| Trpc4 | CGTGGAGCTACTGCTGAACC | CTGGACCGTGAATGCCTGAG |
| Cacna2d1 | AGTGGACCTGGTGCGTATGA | CTCAACTGCCTCAAGGAGCC |
| Trpc1 | GTACACTGCCCCCACCTTTC | GGCGCAGTTCATTGAGGTTC |
| Trpv1 | TCACCGTCAGCTCTGTTGTC | GGGTCTTTGAACTCGCTGTC |
| Scn3a | GGGTGTTGGGTGAGAGTGGAG | AATGTAGTAGTGATGGGCTGATAAGAG |
| Trpm3 | GCCAGGCAAGCCATTCTCTT | CCAGAAGGATGTTCGCCACC |
| Trpm8 | GGCTACACGGTAGGCATTGT | TAAACCGATGCCTCATCTCC |
| Fam38b | TCGCCGTGTTCCAGTTCATC | TTGAAGCAAGTTCTCCCGGC |
